# Supplementary material for: A pan-cancer analysis of homeobox family: expression characteristics and latent significance in prognosis and immune microenvironment
Source: Front Oncol. 2025 Feb 6;15:1521652. doi: 10.3389/fonc.2025.1521652 (PMC11840236; doi:10.3389/fonc.2025.1521652)
Supplement: Supplementary Figure 1 — Mutations of each HOX gene in 32 cancers (TCGA, PanCancer Atlas) by cBioportal. [file DataSheet1.zip › Suppl.files/raw code.zip!/code/4.survival/survivalplot.zip!/survivalplot/HOXA1_OS.CESC.pdf]

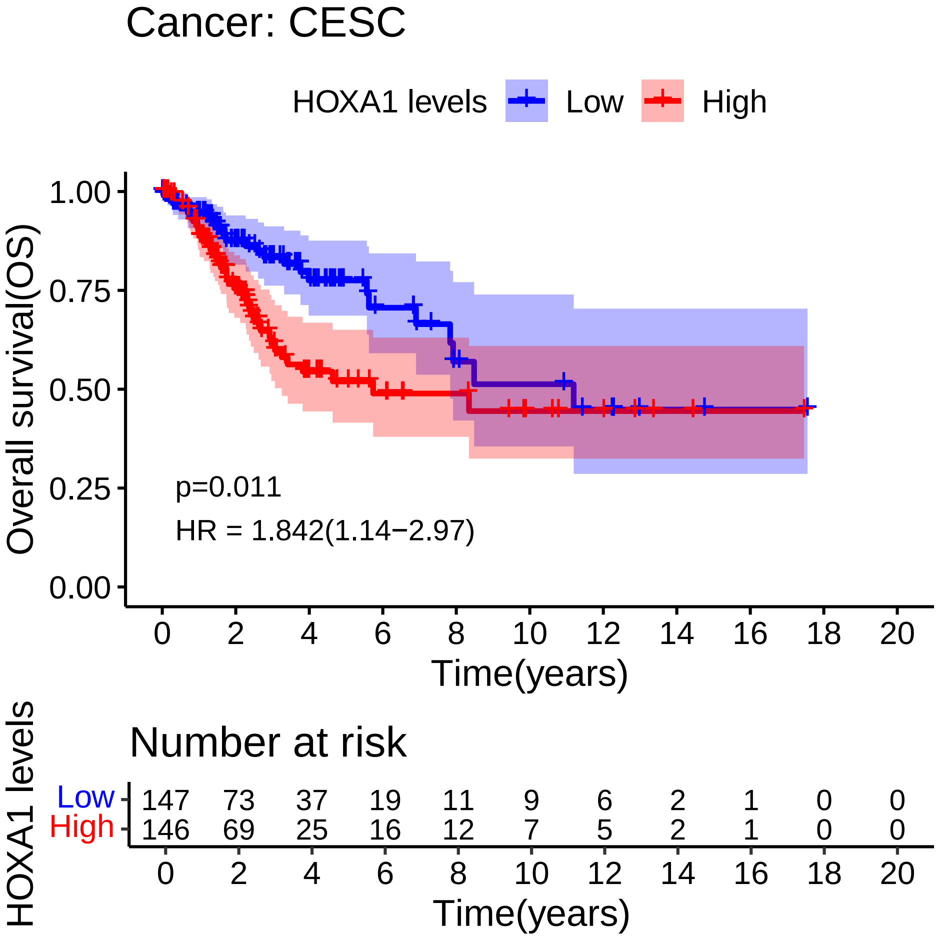
Cancer: CESC
HOXA1 levels
Low
High
Overall survival(OS)
1.00
0.75
0.50
0.25
0.00
p=0.011
HR = 1.842(1.14−2.97)
0 2 4 6 8 10 12 14 16 18 20
Time(years)
Number at risk
HOXA1 levels
Low 147 73 37 19 11 9 6 2 1 0 0
High 146 69 25 16 12 7 5 2 1 0 0
0 2 4 6 8 10 12 14 16 18 20
Time(years)
